# Three-dimensional analysis of the relationship between the structure of maxillary central incisor and the preparation of dental all-ceramic

**DOI:** 10.1371/journal.pone.0209791

**Published:** 2018-12-27

**Authors:** Peng Wang, Fangfang Sun, Qing Yu, Guofeng Wu

**Affiliations:** Department of Prosthodontics, Nanjing Stomatological Hospital, Medical School of Nanjing University, Nanjing, PR China; Texas A&M University College of Dentistry, UNITED STATES

## Abstract

The purpose of this study is to investigate the interrelationship between tooth preparation and dental structure of Chinese maxillary central incisors and provide scientific guidance for clinical all-ceramic restoration. Forty-five specimens of maxillary central incisors were fabricated by 3D printer based on data of Micro CT scanning. Subsequently, every three specimens from the same natural tooth were randomly divided into three groups (n = 15): porcelain veneer, all ceramic crown, and the blank control group. All the specimen teeth were prepared according to routine clinical criteria, reconstructed into 3D models and then measured in software. The results showed that the mean quantity of reduction (volume fraction) was (28.35 ± 4.37) % and (56.93 ± 3.47) % for porcelain veneer and all-ceramic crown, respectively. The bonding areas of different all-ceramic restorations were (128.85 ± 11.73) mm^2^ and (97.15 ± 9.98) mm^2^ for all-ceramic crown and porcelain veneer respectively. In porcelain veneer group, the area of enamel adhesive was (54.80 ± 12.70) mm^2^, and the area of dentin was (42.35 ± 9.62) mm^2^. As the results of the one-sample t test, the mean distances from medullary angle to incisal edge or adjacent surface have significant differences with the test value which was set as 0.5 (P < .05). The reduction of the tooth for porcelain veneer is less than that of ceramic crown and the cementation of porcelain veneer is mostly dependent on the conservation of the enamel during preparation. The region from mesial adjacent surface to mesiopulpal angle is prone to have the problem of medullary perforation.

## Introduction

Dental professionals strive to provide esthetic restorations replacing natural defective teeth, particularly in the anterior region. Aesthetically superior restorations are now mostly applied as a result of the great improvements in the restoration materials and fabrication techniques, whereas, all-ceramic restoration for the incisors is still the most prevalent method in clinical practice [1–3],which includes porcelain veneers [4, 5], all-ceramic crowns and so on. However, the preparation of all-ceramic restoration is still an intractable issue in the clinic which has been accentuated by the social emphasis [6].The preparation of full porcelain restorations are significantly greater than traditional restoration, such as metal ceramic crowns or metal crowns. Unfortunately, till now, few studies were conducted to understand three-dimensional morphology of Chinese teeth, which could guarantee clinically acceptable tooth preparation for all ceramic restorations. The lack of existing knowledge in three-dimensional morphology of Chinese teeth is very easy to cause the pulp injury in the clinic for Chinese patients, resulting in dissatisfaction and great losses to the patients [7].

In the past, the successful tooth preparation and restoration-bonding are dependent on the abundant clinical experience [8]. As the all ceramic restorations are normally applied in the anterior region [3], which contains porcelain veneers and all-ceramic crowns, it is necessary to systematically analyze the three-dimensional morphology of the Chinese teeth, and to master the bonding area about enamel and dentin of anterior teeth after preparation and provide scientific basis for the rational selection of bonding method in clinical practice. Meanwhile, the remaining tooth tissue thickness [7] and other important parameter after preparation should be accurately studied to provide reference for the improvement of clinical preparation.

The aim of the present study is to investigate the interrelationship between tooth preparation and dental structure of Chinese maxillary central incisors and provides scientific guidance for clinical all-ceramic restoration. A digital method by measuring the volume of the tooth reduction before and after preparation was introduced into the present study, which could avoid error generated by the different structures of teeth. The length, area and volume of each tooth structure were accurately measured by digitalized high-tech instruments, which are more accurate, objective and repeatable than the other ways [9, 10].

## Materials and methods

A total of fifteen samples of maxillary central incisors were collected and the donors' personal information (names, addresses etc.) were anonymized. The donors was aged from 20 to 45 years, 7 males and 8 females. Inclusion criteria are as follows: normal crown shape, no dentin exposed, no significant wear, caries-free, un-restored upper left central incisor, no history of root canal treatment, and no tooth surface crack. Teeth with variation of pulp chamber and calcifications were excluded.

**Fig 1:**
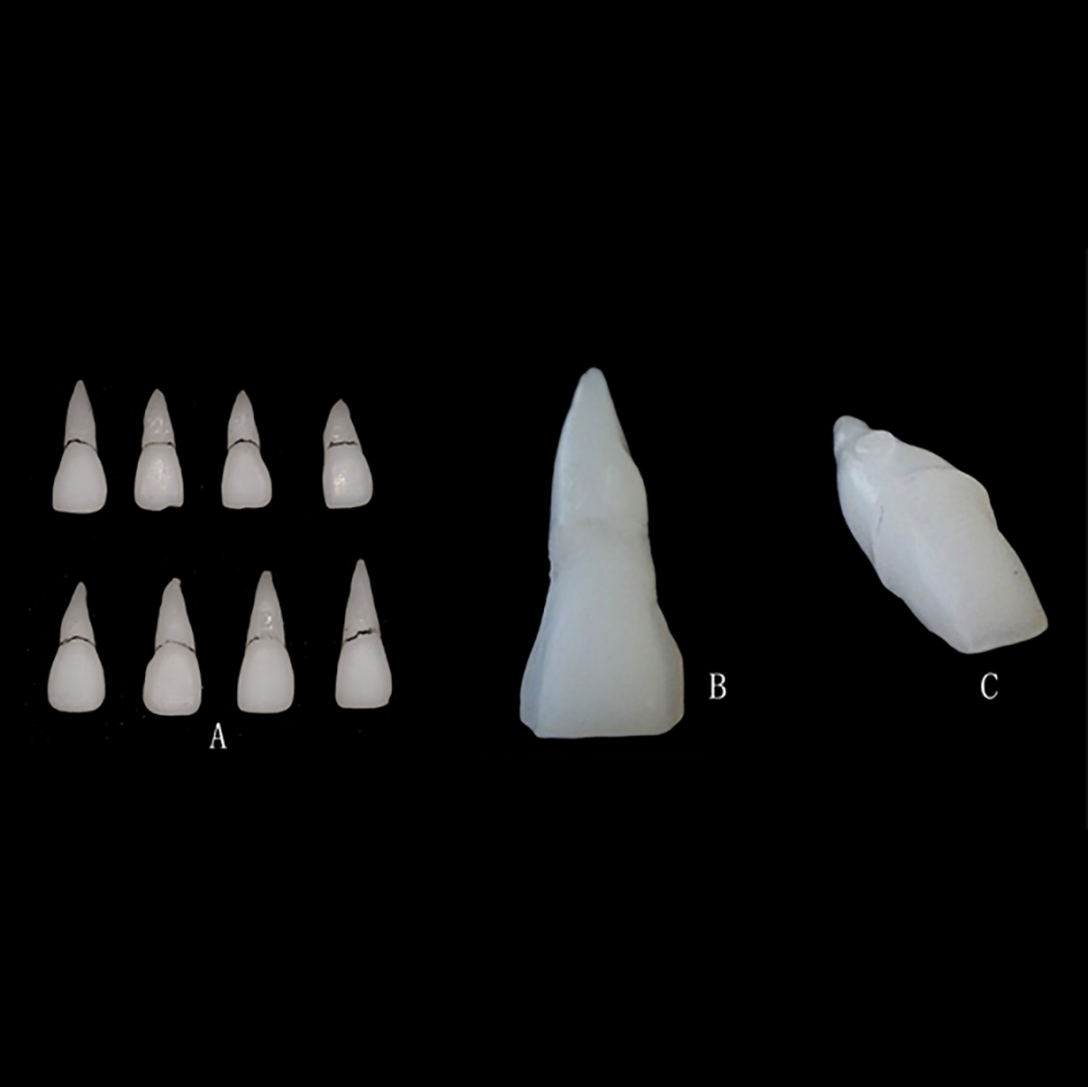
The forty-five specimens which were fabricated by 3D printer. (A) Original specimens; (B) A specimen prepared for porcelain veneer; (C) A specimen prepared for all ceramic crown.

A thorough cleaning for fifteen samples of maxillary central incisors was performed under a magnifying glass. A total of fifteen random samples were scanned by Micro CT (micro-computed tomography, 80Kv, 500μA, 19.64μm, 800ms). All the scan results were expressed in “DICOM” and reconstructed by reverse engineering software (Mimics10.01 and Geomagic Studio11.0). The data of reconstructed three-dimensional model were saved as “STL”, and then printed by Stratasys EDEN 260v 3D printer (Ambient 29, Tray 20°C, Heads vacuum 6.1, Pre-heater 70C). A total of forty-five specimen were fabricated (Fig 1A) with three specimens for each sample of maxillary central incisors. Subsequently, they were randomly divided into three groups (n = 15 each, every three specimen from the same natural tooth were randomly divided into the three groups): group A (A1-A15): porcelain veneer, group B (B1-B15): all ceramic crown, and group C (C1-C15): the blank control group.

All the specimen teeth of group A were prepared according to the routine and standard clinical criteria: minimum 1.2mm to 1.5mm incisal edge, 0.7mm for incisal end, 0.5mm for middle third region, 0.3mm for neck area [11, 12]. Overlapped porcelain laminate veneers were prepared (Fig 1B).

All the specimen teeth of group B were prepared according to the routine and standard clinical criteria: minimum 1–1.5mm for incisal end, minimum 3-degree taper, 1.0mm for all-ceramic shoulder, minimum 0.8–1.5mm for lingual and buccal sides, minimum 1.0–1.5mm for proximal surface [13] (Fig 1C).

All the specimen teeth were prepared by the same dentist with more than five years of clinical experience. Silicone rubber guide plates were used to guarantee that the preparation for group A and B was strictly conformed to the routine clinical criteria.

Micro CT (micro-computed tomography, 80Kv, 500μA, 19.64μm, 800ms) and Mimics software (Mimics10.01 and Geomagic Studio11.0) were used to scan and reconstruct the specimen teeth of group A, group B, and group C after the preparation. Geomagic software was used to measure the tissue amount (volume ratio, V) and the bonding area (mm^2^,) of the prepared specimen teeth. The distance (D) from medullary angle to incisal edge or adjacent surface in group B was measured by the software at the same time. The volumes of the specimen teeth (V_Group_, Group = A, B or C) and the pulp chambers (V_Group_’) from the three groups were measured by the Geomagic software. Every specimen tooth was measured three times and averaged as the final data. All the above measurements were performed by the same dentist.

The volume of the reduction tooth (ΔV) was calculated using the following equation:

$$\Delta V_{A}=V_{C}-V_{A}$$

The volume ratio (α) of the preparation was calculated using the following equation:

$$a_{A}=\frac{\Delta V_{A}}{V_{C}-V_{C^{\prime }}}\times 100\%$$

The paired samples t test was used to analyze the differences of α between the different methods of porcelain restorations. The differences of the bonding areas between the two restore methods or the different hard tissues (enamel and dentine) in group A were analyzed.

The one-sample t test and the paired samples t test were used to analyze the differences of the distances from medullary angle to incisal edge or adjacent surface among group A and B.

The statistical analyses were performed using software (SPSS Package v20.0 for Windows; SPSS Inc).

## Results

**Fig 2:**
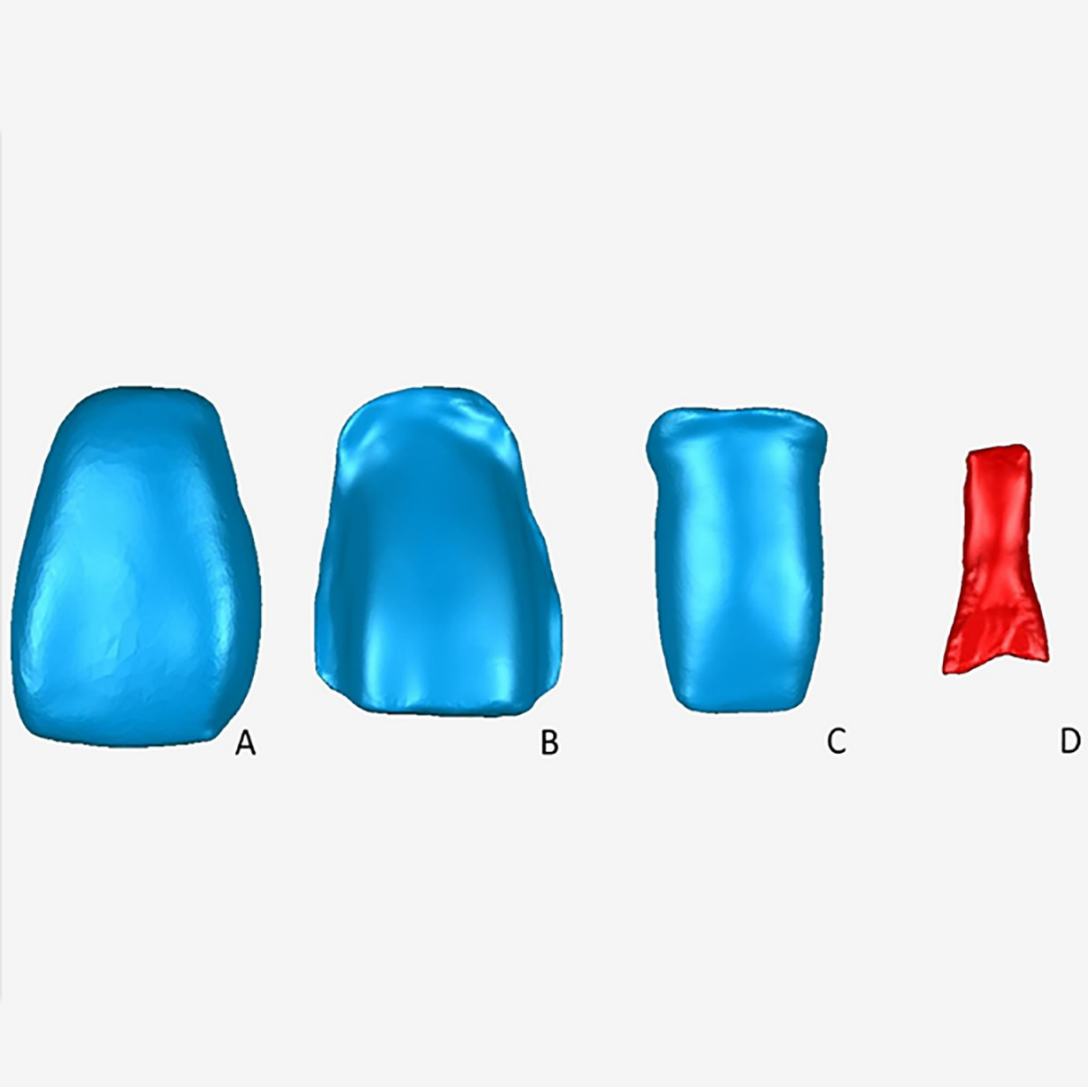
Sketch map for the measurement of specimens of maxillary central incisors after preparation for full-porcelain restoration. (A) The crown of an original specimen; (B) A specimen prepared for porcelain veneer; (C) A specimen prepared for all ceramic crown; (D) Medullary chamber of the crown.

The crown and pulp-chamber of all the specimen teeth were scanned and reconstructed by Micro CT and Mimics software before and after teeth preparation (Fig 2)

The volume ratios (α) of the preparation teeth from group A and B were shown in Table 1.

**Table 1:** The volume ratios (α) of the preparation teeth from group A and B.

	Volume Ratio (α,%)	
No.	Veneers Group (A)	Crowns Group (B)
1	31.56	51.75
2	34.61	62.61
3	35.37	62.35
4	31.6	56.56
5	29.71	52.19
6	22.96	61.11
7	22.11	57.56
8	26.95	53.50
9	25.58	59.44
10	30.47	57.33
11	24.30	57.41
12	25.39	57.83
13	25.44	54.37
14	25.35	56.71
15	33.92	53.18
Mean (standard deviation)	28.35±4.37	56.93±3.47

The α of the preparation teeth from group B (56.93±3.47)% is much larger than that of group A (28.35±4.37)%.

The bonding areas of the two methods of porcelain restorations were shown in Table 2. The mean adhesive area of all-ceramic crown is (128.85 ± 11.73) mm^2^ and that of porcelain veneer is (97.15 ± 9.98) mm^2^. There was significant departure in the bonding area between the two groups (P < .05).

**Table 2:** The bonding areas(mm^2^) of the two methods of porcelain restorations.

	Adhesive Area (mm^2^)	
No.	Veneers Group (A)	Crowns Group (B)
1	91.2	127.06
2	99.26	131.25
3	102.48	136.94
4	103.66	138.37
5	87.42	130.98
6	90.48	129.15
7	104.47	137.12
8	95.34	133.78
9	102.17	139.30
10	90.37	111.29
11	90.84	124.37
12	106.32	125.56
13	89.15	106.49
14	121.83	149.38
15	82.29	111.72
Mean (standard deviation)	97.15±9.98	128.85±11.73

The bonding areas of the different hard tissues (enamel and dentine) in group A were shown in Table 3.

**Table 3:** The bonding areas of enamel and dentine in porcelain veneer group (A).

	Adhesive Area (mm^2^) of Veneers Group (A)	
No.	Enamel	Dentine
1	43.68	51.66
2	42.25	48.95
3	67.62	31.64
4	60.33	42.15
5	39.53	47.89
6	41.48	49.00
7	42.63	61.84
8	70.89	32.77
9	53.61	48.56
10	51.25	31.04
11	54.98	35.86
12	44.28	46.09
13	74.15	32.17
14	59.60	29.55
15	75.77	46.06
Mean (standard deviation)	54.80±12.70	42.35±9.62

The area of enamel adhesive was (54.80 ± 12.70) mm^2^ and that of dentin was (42.35 ± 9.62) mm^2^. According to the result of paired samples t test, the area of enamel adhesive was significantly lager than that of dentin (P < .05)

**Fig 3:**
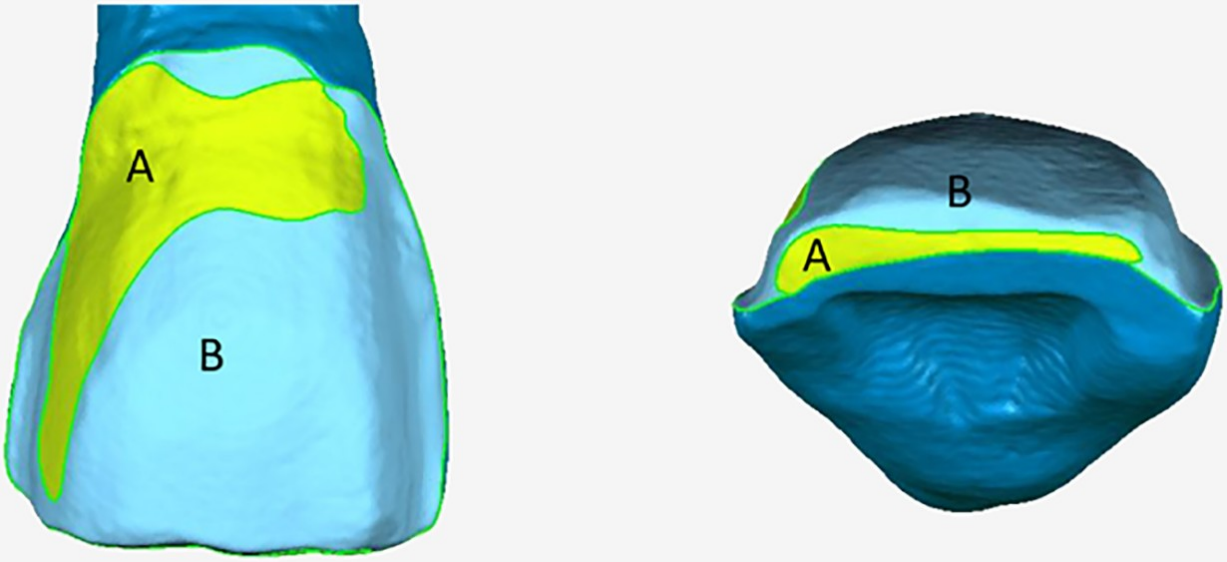
The distribution map of bonding areas of different hard tissue (enamel and dentine) in group A. (A) The region of dentine; (B) the region of enamel.

The enamel and dentine areas of the specimen teeth in group A after veneer preparation were reconstructed and measured by Geomagic Studio11.0 and were presented in Fig 3. The results showed that most dentine bonding areas (Fig 3A) spread near the cervical and incisal region of the teeth.

**Fig 4:**
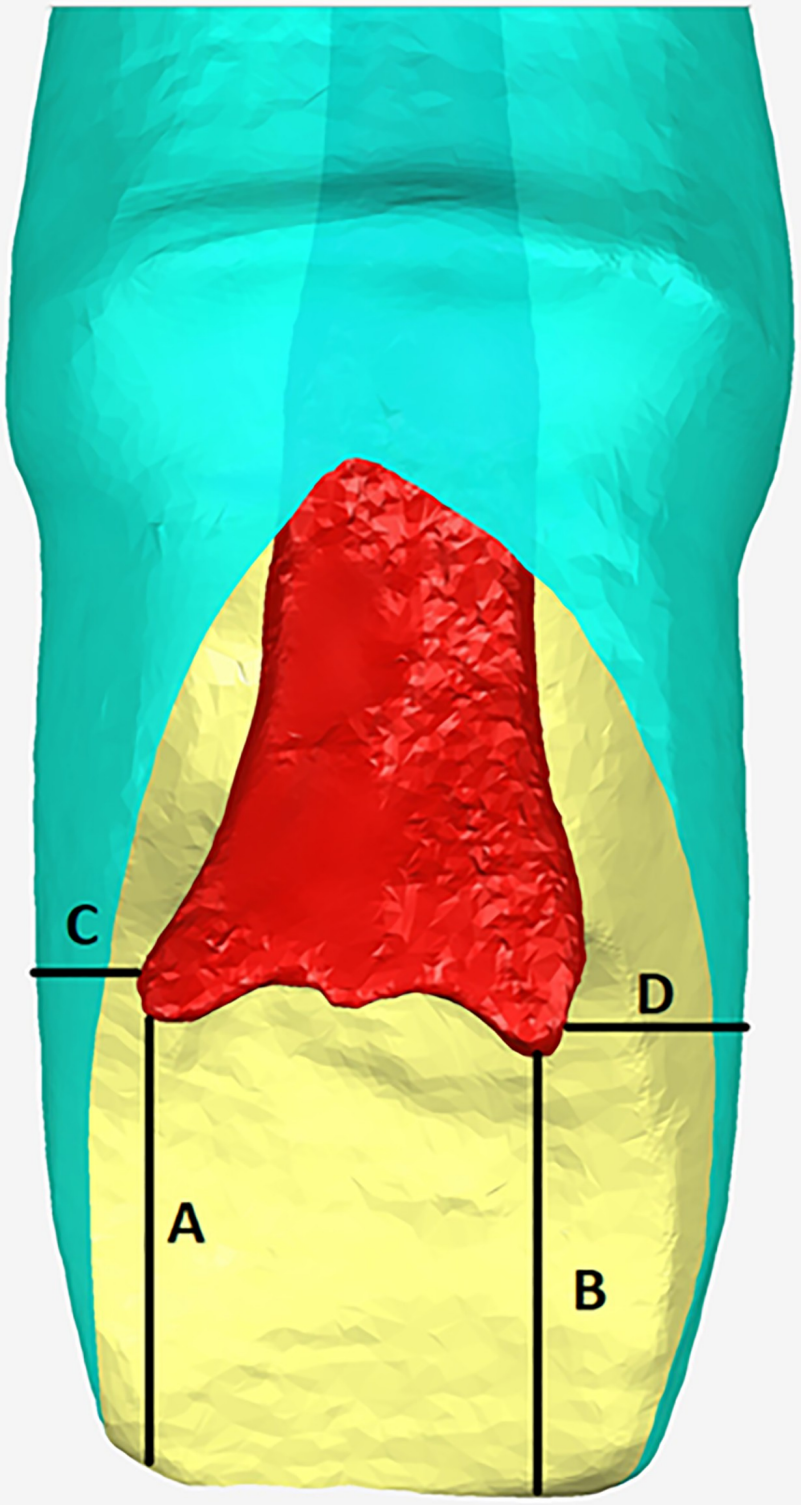
The distance from medullary angle to incisal edge or adjacent surface of the specimen which was prepared for all ceramic crown. (A) The distance from mesiopulpal angle to incisal edge; (B) The distance from distopulpal angle to incisal edge; (C) The distance from mesiopulpal angle to mesial adjacent surface; (D) The distance from distopulpal angle to distal adjacent surface. The red part in the Fig 4 was the medullary chamber of the crown, and the green part was a specimen prepared for all ceramic crown.

The distance from medullary angle to incisal edge or adjacent surface for each specimen tooth in group B were indicated in Fig 4 and recorded in Table 4.

**Table 4:** The distance from medullary angle to incisal edge or adjacent surface for each specimen tooth in group B.

	Thickness of Hard Tissure Remianed in Crowns Group(B)			
No.	D_1_	D_2_	D_3_	D_4_
1	3.5	4.1	1.14	1.72
2	4.52	4.83	1.91	1.19
3	4.17	4.23	1.63	1.48
4	3.55	2.66	1.38	1.02
5	4.65	3.90	1.27	1.51
6	2.30	3.47	0.77	1.30
7	0.74	1.15	0.58	0.89
8	2.17	1.39	0.77	0.68
9	2.32	3.94	1.05	1.99
10	2.22	2.44	0.69	1.50
11	3.75	1.78	0.2	0.68
12	2.63	2.05	0.34	0.74
13	2.44	1.37	0.68	1.35
14	3.35	1.11	1.62	0.87
15	0.53	1.1	0.52	1.13
Mean (standard deviation)	2.86±1.23	2.63±1.33	0.97±0.51	1.20±0.40

The distances from mesiopulpal angle to incisal edge, from distopulpal angle to incisal edge, from mesiopulpal angle to mesial adjacent surface and from distopulpal angle angle to distal adjacent surface were recorded as D_1_, D_2_, D_3_ and D_4_, respectively in Table 4.

## Discussion

In spite of advances in clinical dentistry over the past decades, the methods to measure the quantity of reduction after tooth preparation has remained almost unchanged. The electronic analytical balance is attractive because it allows an objective assessment of the tooth weight independent of uniform density [14]. However, the density was different among the enamel, dentine, cementum and the endodontium. Furthermore, the density changes among different persons and teeth. The measuring method by weighing natural teeth has been well documented. Nevertheless, the digital method by measuring the volume of the reduction tooth after preparation has not been extensively investigated [15].

In this study, we successfully reconstructed a three-dimensional delicate model of human maxillary central incisor preparations: porcelain veneer and all-ceramic crown. The reduction could be calculated for the first time by the following formula: $a_{A}=\frac{\Delta V_{A}}{V_{C}-V_{C^{\prime }}}\times 100\%$. The results showed that porcelain veneer was 28.35 ± 4.37% and all-ceramic crown was 56.93 ± 3.47%. The reduction in all-ceramic crown was significantly higher than that of the porcelain veneer (P < .05) which was a popular mean of correcting aesthetic because of the conservative amount of tooth structure that needs to be removed [16, 17]. Pearson correlation analysis suggested that there was no correlation between the two types of restoration (P> .05). The all-ceramic crown preparation cut much more hard tissue from the maxillary central incisor, indicating that we need a sufficiently large tooth for crown restoration [13] and less hard tissue could be remained leading to the highest risk of pulp injury. Because less hard tissue was remained, service life of the crown becomes shorter.

The three-dimensional model of the dentinal nucleus-root structure of maxillary central incisor was successfully reconstructed. The bonding area of all-ceramic crown and porcelain veneer was 128.85 ± 11.73 mm^2^, and 97.15 ± 9.98 mm^2^, respectively. The bonding area was significantly different between the two groups (P < .05). The smaller bonding area for porcelain veneer after preparation suggested that bonding method for porcelain veneers is important in clinical practice.

In group A (porcelain veneer group), the area of enamel adhesive was (54.80 ± 12.70) mm^2^, and the area of dentin was (42.35 ± 9.62) mm^2^. According to the result of paired samples t test, the area of enamel adhesive was significantly lager than that of dentin (P < .05), indicating that the exposed enamel area was greater than dentin after porcelain veneers preparation. Large exposed enamel tissue area suggests that enamel adhesives should be better [18–20] or cemented according to different tissues in clinical dentistry. Friedman et al [21] reported that the bonding of all porcelain veneers should be depended on the enamel areas, which is consistent with this study. Ayoub et al reported that tooth preparation for porcelain veneers should be interenamel to maximize the resin bond strength and the resin bonding is a quite reliable and predictable method in the enamel [22]. The results (Table 3 and Fig 3) of this study indicated that it is very important to guarantee the conservation of the enamel during preparation of the tooth for porcelain veneer [22–24].

Previous studies [7] have measured and analyzed 16 maxillary central incisors prepared for the metal ceramic crowns by Micro CT. The residual dentin thickness was displayed by three-dimensional color-coded graph. The results are intuitive and accurate. However, the teeth in vitro have been destroyed in the process of study and thus the experiments cannot be re-performed. With respect to the upright range between pulp chamber and leftover dentine, the distance (Fig 4) from mesiopulpal angle to incisal edge (D_1_), distopulpal angle to incisal edge (D_2_), mesiopulpal angle to mesial adjacent surface (D_3_) and distopulpal angle angle to distal adjacent surface (D_4_) were (2.86 ± 1.23) mm, (2.63 ± 1.33) mm, (0.97 ± 0.51) mm, and (1.20 ± 0.40) mm, respectively. The mean distance from pulp chamber to incisal edge was higher than the distance from pulp chamber to adjacent surface. The difference between the mesial or the distal medullary angle to the cutting incisal edge was not statistically significant (P>.05), the difference between the mesial or the distal medullary angle to the adjacent surface was not statistically significant (P>.05) and there were significant differences between the other groups (P < .05).

Murray et al [25] reported that the dentine should be remained with at least 0.5mm thickness after tooth preparation, because the damaged dental pulp can be repaired by itself within this range. And Camps et al [26] also reported that the highest risk of pulp injury would occur when the thickness of the remaining dentine tissue was less than 1 mm. With respect to the result of the one-sample t test, the mean distance of the four groups (D_1_, D_2_, D_3_, D_4_) has significant difference (test value = 0.5). However, the mean distance (D_3_) from mesiopulpal angle to mesial adjacent surface has no significant difference (P = .60, test value = 0.9). The other mean distances (D_1_, D_2_, D_4_) were significantly longer than 0.9 mm (P < .05). According to previous reports, a shorter distance from mesial pulp angle to mesial adjacent surface would resulted in the highest risk of pulp injury much easily [7]. More attention should be paid during the preparation around the above-mentioned region of maxillary central incisors in clinical practice.

## Conclusion

This study introduced a digital approach for investigating the interrelationship between tooth preparation and dental structure of the maxillary central incisors. The data including the volume, the area and the length among the different tooth structures after preparation obtained in this study provided scientific guidance for clinical all-ceramic restoration in clinic. The highest reduction was observed in all-ceramic crown among other all-ceramic restorations, which resulted in the highest risk of pulp injury. Exposed enamel area was greater than dentin for maxillary central incisor after porcelain veneers preparation. Dentin tissue was distributed over the dental neck and the cut edge of the tooth, suggesting that universal adhesives or bonding should be used according to different hard tissue.
